# Supplementary material for: Structure and Nanomechanics of PPTA-CNT Composite Fiber: A Molecular Dynamics Study
Source: Nanomaterials (Basel). 2022 Sep 9;12(18):3136. doi: 10.3390/nano12183136 (PMC9504707; doi:10.3390/nano12183136)
Supplement: Supplementary file 1 [file nanomaterials-12-03136-s001.zip › nanomaterials-1881238-supplementary-done.pdf]

nanomaterials

MDPI

Article

Structure and Nanomechanics of PPTA-CNT Composite Fiber: A Molecular Dynamics Study

Tong Li *,†, Zebei Mao †, Juan Du [1] and Zhuoyu Song

Department of Engineering Mechanics, Dalian University of Technology, Dalian 116024, China
* Correspondence: tong@dlut.edu.cn; Tel.: +86-411-84706036
† These authors contributed equally to this work.

The force field parameters used in this paper are summarized in the following tables, which are 9-6 Lennard–Jones interaction, bond interaction, angle interaction, and dihedral interactions.

The derivation of force field parameters of the new CG molecules usually requires the inverse Monte Carlo method or the force-matching method to complete the parameterization after comparing with the all-atom simulation [1]. Multiple systems were created to obtain the calculation parameters based on the simulation results, including the bond interactions, angle interactions, dihedral interactions, and non-bonding interactions. The 9-6 Lennard–Jones interaction parameters are shown in Table S1, except for the interaction parameters of the N-O pair ($\varepsilon = 0.5393$ and $\sigma = 3.6246$). The bond interaction parameters are shown in Table S2. The angle interaction parameters are shown in Table S3. The dihedral interaction parameters are shown in Table S4.

Table S1. Force field parameters of the 9-6 Lennard–Jones interaction of CG particles.

Particle type	Mass	ε (kcal/mol)	σ (Å)
BR1	12.01	0.1750	3.8000
BR2	26.04	0.1750	3.8000
BR3	26.04	0.1750	3.8000
BR4	12.01	0.1750	3.8000
N	15.01	0.2430	4.0506
O	28.01	0.3233	3.7880
H_2SO_4	98.08	0.9800	4.2700

Table S2. Force field parameters of the bond interaction of CG particles.

type i	type j	k_r (kcal/mol/Å^2)	r_0 (Å)
BR1	BR2	40.0	1.911
BR1	BR3	40.0	1.910
BR1	N	40.0	1.447
BR1	O	30.0	1.949
BR2	BR4	40.0	1.910
BR3	BR4	40.0	1.910
BR4	N	40.0	1.447
BR4	O	30.0	1.949
N	O	36.0	1.872

Table S3. Force field parameters of the angle interaction of CG particles.

type i	type j	type k	$k_{\theta,1}$ (kcal /mol/rad^2)	$\theta_{0,1}$ (Å)	$k_{\theta,1}$ (kcal /mol/rad^2)	$\theta_{0,2}$ (Å)
BR1	BR2	BR4	462.0	95.6	0.0	0.0
BR1	BR2	COOH	129.0	137.0	0.0	0.0
BR1	N	O	75.0	104.6	0.0	0.0
BR1	O	N	214.0	79.5	0.0	0.0
BR1	BR3	BR4	462.0	95.6	0.0	0.0
BR2	BR1	BR3	445.0	84.4	0.0	0.0
BR2	BR1	N	93.0	131.8	88.0	143.2
BR2	BR1	O	127.0	118.5	96.0	156.0
BR2	BR4	BR3	445.0	84.4	0.0	0.0
BR2	BR4	N	93.0	131.8	88.0	143.2
BR2	BR4	O	127.0	118.5	96.0	156.0
BR3	BR1	N	93.0	131.8	88.0	143.2
BR3	BR1	O	127.0	118.5	96.0	156.0
BR3	BR4	N	93.0	131.8	88.0	143.2
BR3	BR4	O	127.0	118.5	96.0	156.0
BR4	N	O	75.0	104.6	0.0	0.0
BR4	O	N	214.0	79.5	0.0	0.0

Table S4. Force field parameters of the dihedral interaction of CG particles.

type i	type j	type k	type l	k_1	k_2	k_3	k_4
BR1	BR2	BR4	BR3	0.000	14.375	0.000	0.000
BR1	BR2	BR4	N	10.000	0.000	0.000	0.000
BR1	BR2	BR4	O	5.000	0.000	0.000	0.000
BR1	BR3	BR4	BR2	0.000	14.375	0.000	0.000
BR1	BR3	BR4	N	10.000	0.000	0.000	0.000
BR1	BR3	BR4	O	5.000	0.000	0.000	0.000
BR1	N	O	BR4	33.100	0.000	0.000	0.000
BR2	BR1	BR3	BR4	0.000	14.375	0.000	0.000
BR2	BR1	N	O	0.084	0.364	0.068	-0.236
BR2	BR1	O	N	-0.148	0.620	0.272	-0.026
BR2	BR4	N	O	0.084	0.364	0.068	-0.236
BR2	BR4	O	N	-0.148	0.620	0.272	-0.026
BR3	BR1	BR2	BR4	0.000	14.375	0.000	0.000
BR3	BR1	N	O	0.084	0.364	0.068	-0.236
BR3	BR1	O	N	-0.148	0.620	0.272	-0.026
BR3	BR4	N	O	0.084	0.364	0.068	-0.236
BR3	BR4	O	N	-0.148	0.620	0.272	-0.026
BR4	N	O	BR1	33.100	0.000	0.000	0.000
N	BR1	BR2	BR4	10.000	0.000	0.000	0.000
N	BR1	BR3	BR4	10.000	0.000	0.000	0.000
O	BR1	BR2	BR4	5.000	0.000	0.000	0.000
O	BR1	BR3	BR4	5.000	0.000	0.000	0.000

Reference

1. Mogurampelly, S.; Macdermaid, C.M.; Percec, S.; Klein, M.L.; Fiorin, G. Aggregation of poly (p-phenylene terephthalamide) chains: Emergence of fiber defects. *Phys. Rev. Mater.* **2019**, *3*, 015602.
